# Prevalencia de eventos adversos y sus manifestaciones en profesionales de la salud como segundas víctimas

**DOI:** 10.7705/biomedica.6169

**Published:** 2022-03-01

**Authors:** Fredy Flórez, Lucelly López, Catalina Bernal

**Affiliations:** 1 Facultad de Medicina, Universidad Pontificia Bolivariana, Medellín, Colombia Universidad Pontificia Bolivariana Universidad Pontificia Bolivariana Medellín Colombia

**Keywords:** near miss salud, personal de salud, errores médicos, seguridad del paciente, apoyo social, Near miss, healthcare, health personnel, medical errors, patient safety, social support

## Abstract

**Introducción.:**

Los eventos adversos pueden causar daño al paciente y también afectar a los profesionales de la salud, lo que los convierte en segundas víctimas. Las intervenciones se han enfocado principalmente en los pacientes, pero poca atención se ha prestado a los profesionales de la salud, probablemente por falta de herramientas.

**Objetivo.:**

Estimar la prevalencia de eventos adversos y describir sus manifestaciones en el personal asistencial, con el fin de evidenciar el fenómeno de las segundas víctimas en un hospital de alta complejidad.

**Materiales y métodos.:**

Se hizo un estudio transversal analítico mediante una encuesta a 419 profesionales asistenciales de las áreas de hospitalización, urgencias y cirugía en un hospital de alta complejidad de Medellín en el 2019. Se estimó la frecuencia de eventos adversos, y se determinó su asociación con algunas variables laborales y demográficas.

**Resultados.:**

El 93,1 % de los entrevistados conocía de casos de incidentes y el 79 %, de eventos adversos graves. El 44,4 % se había visto involucrado en un evento adverso, y el 99 % de estos expresaba sentirse como segunda víctima por experimentar dificultad para concentrarse, sentimientos de culpa, cansancio, ansiedad y dudas sobre sus decisiones. El 95 % quería recibir capacitación para afrontar las consecuencias de los eventos adversos y saber cómo informar al paciente.

**Conclusiones.:**

Con frecuencia los profesionales de la salud se exponen a eventos adversos que pueden causarles emociones negativas como culpa, cansancio, ansiedad e inseguridad. La mayoría de los profesionales que participan en un evento adverso manifiestan sentimientos como segunda víctima. El informar al paciente sobre un evento adverso requiere preparación y la mayoría de los profesionales entrevistados pidió capacitación en el tema.

## Abstract (en)

**Introduction:**

Adverse events can cause harm to the patient, but they also affect health professionals making them second victims of the event. Interventions have been focused mostly on patients, but little on professionals, probably due to lack of tools.

**Objective:**

To estimate the prevalence of adverse events and describe their manifestations in healthcare personnel to evidence the phenomenon of second victims in a highly complex hospital.

**Materials and methods:**

We conducted an analytical cross-sectional study by a survey of 419 healthcare professionals from the hospitalization, emergency, and surgical areas in a highly complex hospital in Medellín in 2019. The frequency of adverse events was estimated and its association with some labor and demographic variables was determined.

**Results:**

We found that 93.1% of the participants knew of incident cases and 79% of serious adverse events while 44.4% had been involved in them and 99% of these had feelings as a second victim, mainly the difficulty to concentrate, guilt, fatigue, anxiety, and doubts about decisions; 95% indicated they wanted to receive training to face the consequences of adverse events and know how to inform the patient.

**Conclusions:**

Health professionals are frequently exposed to adverse events that can cause negative emotions in them such as guilt, fatigue, anxiety, and insecurity. Most professionals who participate in an adverse event express feelings as a second victim. Informing the patient about an adverse event requires preparation and most professionals requested training on the subject.

Los eventos adversos son el resultado de una atención en salud que produce daño no intencional al paciente, y se pueden clasificar en prevenibles y no prevenibles. Los prevenibles se dan cuando se produce un resultado no deseado, no intencional, que se habría evitado mediante el cumplimiento de los estándares del cuidado asistencial disponibles en un momento determinado, y los no prevenibles cuando, a pesar del cumplimiento de los estándares, se presentan estos resultados indeseados y no intencionales ^1^.

La seguridad del paciente cobró importancia especialmente desde que, en el 2005, la Organización Mundial de la Salud (OMS) estableció directrices en este sentido y creó la Alianza Mundial para la Seguridad del Paciente, con el fin de fomentar la adopción de políticas frente a temas como la notificación y el aprendizaje a partir de errores para mejorar la seguridad en la atención. En el 2009, se publicó un marco conceptual para unificar conceptos referidos a la seguridad del paciente y presentar el ciclo continuo de aprendizaje, el cual incluye desde información sobre los tipos de incidente hasta medidas para disminuir el riesgo ^2^.

En Colombia, las normas vigentes incluyen la Resolución 3100 de 2019 sobre la habilitación de las instituciones prestadoras de servicios de salud y su obligatoriedad de contar con una política de seguridad del paciente que garantice la medición, el análisis, la gestión y el reporte de los eventos adversos, además de crear una cultura de seguridad del paciente con un enfoque educativo mas no punitivo ^3^. Por otro lado, el Decreto 1011 de 2006 señala que toda institución de salud debe contar con un programa de auditoría para el mejoramiento de la calidad que incluya la alerta, la obligación de informar y el análisis de los eventos adversos ^4^. Asimismo, el manual de acreditación regulado por la Resolución 5095 de 2018, exige la implementación de una política de seguridad del paciente en la que se incluya el seguimiento de los eventos adversos, su reporte, análisis y gestión, así como la mitigación de sus consecuencias ^5^.

A nivel mundial, a diario se presentan eventos adversos en las instituciones de salud y, según la OMS, uno de cada diez pacientes que ingresa a un hospital sufre algún tipo de daño a causa de la atención ^6^. En un estudio realizado en Latinoamérica, se registró una incidencia de este tipo de eventos del 19,8 % y, específicamente en Colombia, la prevalencia fue del 13,1 % ^7^. En otro estudio en un hospital de tercer nivel de complejidad en el país, se encontró que el 34 % de los pacientes incluidos en la investigación había tenido algún tipo de evento adverso durante la atención ^8^, en tanto que, en España, hasta el 57,8 % de los profesionales de la salud se ha visto expuesto en algún momento a ellos ^9^ y, en Estados Unidos, se encontró una incidencia entre cirujanos generales del 90,4 % ^10^. Con base en estos datos, se puede presumir que el número de profesionales de la salud que se ven expuestos a un evento adverso es alto.

El daño grave a un paciente es algo que el personal asistencial no quiere que suceda durante la atención y se sabe que la mayoría de los eventos adversos son el resultado de malos sistemas y no de malas personas ^11^. Además, cuando ocurren, a menudo las instituciones de salud enfocan sus esfuerzos en resarcir el daño que se causó al paciente y en investigar las posibles causas del evento, con el fin de prevenir nuevas apariciones, pero no tienen presente que dichos incidentes causan daño no solo al paciente sino que pueden llegar a causar trastornos en la vida laboral, familiar y personal de los profesionales de la salud involucrados ^9^. Esto los convierte en segundas víctimas del evento adverso, término que se emplea para referirse a los profesionales afectados ^12^. Entre las manifestaciones que presentan las segundas víctimas está depresión, estrés postraumático, ira, sentimiento de culpa y abandono de la profesión ^13^^,^^14^.

En un estudio realizado en España, se evidenció que el 72,5 % de los profesionales de la salud en el área hospitalaria había tenido que enfrentar una experiencia como segunda víctima ^9^. Una revisión sistemática sobre el tema incluyó un estudio en Estados Unidos con 2.500 otorrinolaringólogos, en el que se evidenció una prevalencia de segundas víctimas del 10,4 % y otro con 402 profesionales entre enfermeras, médicos y farmaceutas en el cual la prevalencia fue del 43,3 % ^13^. En Colombia, no se registran datos que permitan un acercamiento al problema de las segundas víctimas de un evento adverso en salud, lo que impide conocer la magnitud del problema en el contexto local.

Conocer la frecuencia con la que el personal de salud se ve expuesto a un evento adverso y los efectos que estos producen en ellos, así como algunas variables que se asocian con la exposición, brindaría herramientas para un análisis enfocado no solo en las necesidades de los pacientes, sino, también, en las del personal de salud que se ve involucrado; además, abriría el camino para que los responsables de las decisiones en el sector procuren la implementación de medidas que resarzan el daño del personal expuesto.

En este contexto, el objetivo del presente estudio fue estimar la prevalencia de eventos adversos y describir sus manifestaciones en el personal asistencial para, así, evidenciar el fenómeno de las segundas victimas en un hospital de alta complejidad.

## Materiales y métodos

Se hizo un estudio transversal analítico entre profesionales de la salud del área asistencial de una institución de alta complejidad en Medellín contratados directamente por la institución y que habían laborado en los servicios de urgencias, hospitalización o cirugía durante el año anterior. No se establecieron criterios de exclusión.

Se obtuvo el aval del Comité de Ética de Investigación en Salud de la Universidad Pontificia Bolivariana, así como del Comité de Ética del área de investigación del Hospital Universitario San Vicente Fundación. Se explicó el estudio a cada uno de los participantes y se obtuvo su consentimiento informado por escrito; las encuestas se hicieron de forma anónima, con el fin de garantizar la confidencialidad de los datos.

El tamaño de muestra se calculó con el programa Epidat 4.2 a partir de un universo de 1.441 profesionales asistenciales. Se usó una frecuencia esperada de eventos adversos del 60 %, un error de estimación absoluto del 4 % y un intervalo de confianza del 95 %, lo que arrojó una muestra de 412.

El muestreo fue estratificado con asignación proporcional de la siguiente forma: 107 médicos entre generales, especialistas y subespecialistas, 77 enfermeras profesionales y 228 auxiliares de enfermería. La captación de las personas se hizo en las reuniones grupales que se realizan por áreas con cierta periodicidad.

Se indagó sobre las características demográficas de la población (sexo, profesión, área de trabajo, edad, tiempo de experiencia profesional), y se aplicó en su totalidad el cuestionario utilizado en el estudio *The aftermath of adverse events in Spanish primary care and hospital health professionals*^9^ sin introducirle ningún cambio o traducirle, ya que se encuentra disponible en español.

Este cuestionario mide la percepción de algunos aspectos del programa de seguridad del paciente, así como del apoyo recibido luego de verse expuesto a un evento adverso. Las posibilidades de respuesta se definieron en una escala de tipo Likert como totalmente de acuerdo, de acuerdo, totalmente en desacuerdo, en desacuerdo, ni de acuerdo ni en desacuerdo, y no sabe no responde. En cuanto a la percepción de la probabilidad de un evento adverso con consecuencias graves para el paciente, las respuestas eran alta, mediana, baja, o ninguna. Las opciones de respuesta para las preguntas referentes a las consecuencias de informar a un paciente sobre el evento, el interés de recibir capacitación sobre temas específicos y el involucramiento directo o no en los eventos, fueron sí o no. Se utilizó una escala para indagar acerca de la percepción de la frecuencia de ciertas acciones en caso de que se presentara un evento adverso, la cual iba de 0 (nada frecuente) a 10 (muy frecuente). Además, se indagó acerca de las sensaciones posteriores al evento adverso, cuyas opciones de respuesta eran nunca, alguna vez, casi siempre, siempre.

La información se recolectó en las reuniones semanales o mensuales a las que asiste la mayor parte del personal por área. En ellas se explicó que la encuesta debía responderse de forma individual. Para controlar el sesgo de memoria se indagó sobre la experiencia de cada cual teniendo en cuenta intervalos de tiempo según la intención de las preguntas, por lo que al iniciar cada bloque de preguntas se definía si se refería al año anterior o a los últimos cinco años. No se indagó en detalle sobre los eventos adversos vividos; para controlar el sesgo de información, se aplicaron las encuestas sin pedir datos que permitieran la identificación de las personas. La información se tabuló en Excel y se importó a los programas SPSS 25.0™ y Stata 15™ para su análisis estadístico.

Para la descripción de las variables cualitativas y la estimación de la frecuencia de los eventos adversos, se usaron frecuencias absolutas y relativas, y en el caso de las variables cuantitativas, se usaron la mediana y los percentiles 25 y 75. Para determinar la asociación entre la presencia del evento adverso, las variables demográficas y laborales y algunas percepciones, se usó la prueba de ji al cuadrado, en tanto que, para variables cuantitativas como la edad y el tiempo de experiencia, se usó la prueba U de Mann-Whitney.

Para ajustar las variables asociadas con la presencia de un evento adverso, se estimó un modelo lineal generalizado con distribución de Poisson y función de enlace logarítmica; el ajuste de los errores se hizo por agrupación de área y a partir de esta se estimaron las razones de prevalencia con sus intervalos de confianza.

Se consideró que había diferencias estadísticamente significativas cuando el valor de p era menor a 0,05.

## Resultados

Se hizo la encuesta a 419 profesionales asistenciales en un hospital de alta complejidad de Medellín. El 75,9 % de ellos correspondió a mujeres, el 50 % tenía una edad de 32 años o menos, y tres de cada cuatro pertenecían al personal de enfermería. El 52,7 % trabajaba en el área de hospitalización y la mitad contaba con una experiencia laboral de nueve años o más (cuadro 1).

**Figura 1:**
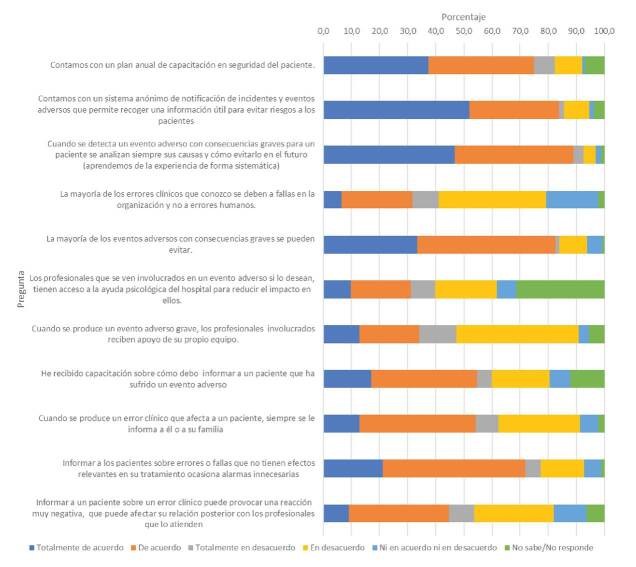
Percepción sobre la seguridad del paciente y manejo de eventos adversos en la institución

En lo referente a la percepción sobre el programa de seguridad del paciente en el hospital, tres de cada cuatro personas expresaron, con diversos grados de acuerdo, que se contaba con un plan de capacitación anual en seguridad del paciente; el 84 % dijo que había un sistema anónimo de notificación de eventos adversos y el 89 % manifestó que cuando se detectaba un evento adverso se analizaban sus causas y las formas de prevenirlo en el futuro. En cuanto al apoyo recibido por parte de la institución después de un evento adverso, el 31 % dijo que los profesionales que se veían involucrados tenían acceso a ayuda psicológica si así lo deseaban (figura 1).

Cuando se les preguntó sobre la experiencia de informar a un paciente de un evento adverso, el 55 % dijo que siempre se informaba a los pacientes o sus familias (figura 1) y tres de cada cuatro consideraban que los pacientes siempre habían aceptado las explicaciones. Sin embargo, el 54 % dijo que informar a un paciente sobre errores que no tienen efectos relevantes en su tratamiento ocasiona alarmas innecesarias, y el 72 % pensaba que informar a un paciente sobre un error clínico puede provocar una reacción negativa que afecta su relación posterior con los profesionales que lo atienden (figura 1). En este sentido, una tercera parte manifestó que, cuando se había informado a los pacientes sobre un efecto adverso, la relación con estos había empeorado. El 57 % refirió no haber recibido capacitación sobre cómo informar a un paciente afectado por un evento adverso (figura 1) y el 95,7 % dijo querer capacitación sobre este tema. Una tercera parte consideró que los profesionales que se ven involucrados en un evento adverso tenían acceso a ayuda psicológica por parte de la institución y un poco menos de la mitad dijo que recibían apoyo de su equipo de trabajo (figura 1).

**Cuadro 1:** Características demográficas y laborales del personal asistencial participante

Característica		n	%
Sexo			
	Hombre	101	24,1
	Mujer	318	75,9
Edad en años [Me (P25 -P75)]		32 (28- 39)	
Profesión			
	Médico general	28	6,7
	Especialista	62	14,8
	Subespecialista	17	4,1
	Enfermera	83	19,8
	Auxiliar de enfermería	229	54,7
Área de trabajo			
	Hospitalización	221	52,7
	Urgencias	144	34,4
	Cirugía	54	12,9
Tiempo de experiencia profesional en años [Me (P25 - P75)]		9 (4 -14)	

*Me: mediana; P25: percentil 25; P75: percentil 75

Las dificultades que más frecuentemente deben afrontar los involucrados (en una escala de 0 a 10) en un evento adverso fueron el miedo a afrontar consecuencias legales y a perder el prestigio profesional, con una calificación por encima de siete en el 75 % de los casos. Asimismo, el 75 % de los participantes dio un puntaje por encima de cuatro en cuanto a la frecuencia con la que se le informa al paciente y a su familia sobre el error y a la petición de disculpas.

Cuando se preguntó por la experiencia en los últimos cinco años, el 93 % informó que había conocido casos de incidentes en el hospital que podrían haber causado daño a un paciente y el 79 % dijo haber conocido casos de eventos adversos con graves consecuencias para el paciente. Por otro lado, tres de cada cuatro personas informaron que habían conocido casos de profesionales con alteraciones en su estado emocional debido al evento adverso de un paciente y el 70,9 % había conocido casos de profesionales que habían tenido problemas laborales debido a un evento de este tipo; una tercera parte manifestó haber tenido que informar personalmente sobre un evento adverso (cuadro 2).

**Cuadro 2:** Positividad de las experiencias sobre eventos adversos propios o ajenos (n=419)

Experiencia en los últimos cinco años	n	%
He conocido casos en mi hospital de situaciones que podrían considerarse “casi errores” (incidentes que podrían haber causado daño a un paciente, pero que finalmente se corrigieron a tiempo).	390	93,1
He conocido casos de eventos adversos con graves consecuencias para uno o varios pacientes.	331	79,0
He conocido casos de profesionales que han estado en mal estado emocional debido a un evento adverso de un paciente.	320	76,4
He conocido casos de profesionales que han tenido problemas laborales debido a un evento adverso.	297	70,9
Personalmente, he tenido que informar a un paciente (o su familia) que ha sufrido un evento adverso.	124	29,6

**Figura 2:**
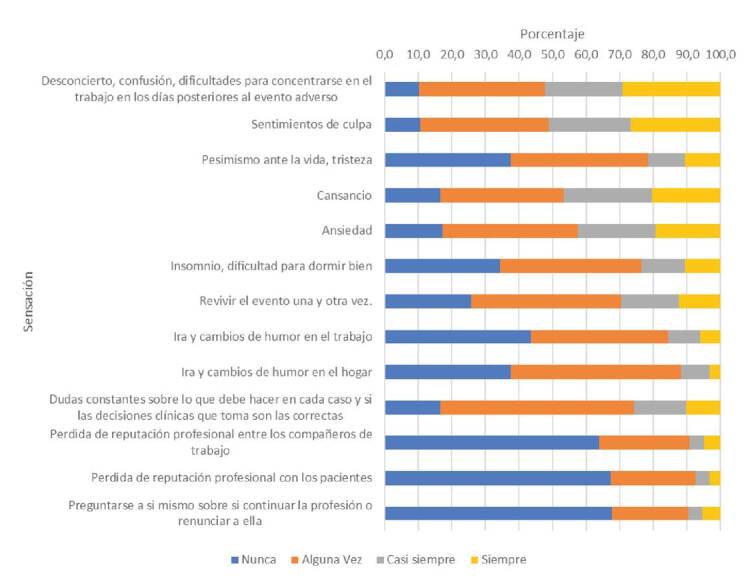
Sensaciones de las personas con respecto a ser segundas víctimas

Por otro lado, el 44 % de los participantes manifestó que se había visto directamente involucrado en un evento adverso y el 99 % de estos manifestó haber tenido, en algún momento después del evento, por lo menos una de las siguientes sensaciones: desconcierto, confusión, dificultades para concentrarse en el trabajo, sentimientos de culpa, cansancio, ansiedad, rememoración del evento una y otra vez, y dudas constantes sobre si sus decisiones clínicas eran las correctas (figura 2).

**Figura 3:**
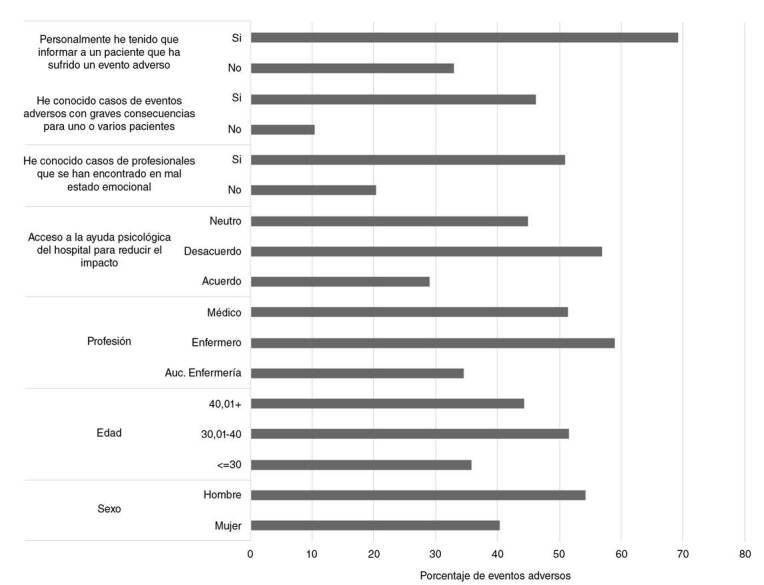
Relación de características sociodemográficas y algunas percepciones ante el hecho de haberse visto directamente involucrado en un evento adverso

En la figura 3, se presenta la relación de algunas características demográficas y percepciones con el hecho de haberse visto directamente involucrado en un evento adverso. Se constató que el porcentaje de personas que se vio involucrado en un evento adverso fue más alto en hombres, en mayores de 30 años, en enfermeros profesionales, y entre quienes han tenido que informar personalmente sobre el evento.

Empleando un modelo lineal, se evidenció que la posibilidad de estar directamente involucrado en un evento adverso aumentaba con la edad, pues fue superior en los mayores de 30 años y en los profesionales de enfermería. Además, se observó que la posibilidad de estar directamente involucrado en el evento era mayor en las personas que manifestaron estar en desacuerdo o ser neutrales cuando se les preguntó por su percepción sobre el acceso a la ayuda psicológica después del evento y en las que manifestaron haber conocido casos de profesionales emocionalmente afectados a causa de este. También, se encontró que dicha posibilidad era mayor en personas que habían sabido de eventos adversos graves y en aquellas que habían tenido que informar personalmente sobre el evento (cuadro 3).

**Cuadro 3:** Asociación de algunos factores con la probabilidad de verse involucrado directamente en un evento adverso

Variable		RPc (IC_95%_)	^p^	RPa (IC_95%_)	^p^
Sexo	Mujer	1		1	
	Hombre	1,35 (1,04-1,77)	0,026	1,09 (0,97-1,22)	0,162
Edad	≤30	1		1	
	30,01-40	1,44 (1,31-1,57)	0,000	1,27 (1,02-1,59)	0,029
	40,01 y más	1,23 (0,95-1,59)	0,109	1,25 (0,84-1,87)	0,264
Profesión	Auxiliar de enfermería	1		1	
	Enfermero	1,71 (1,62-1,81)	0,000	1,34 (1,28-1,41)	0,000
	Médico	1,49 (1,25-1,78)	0,000	0,90 (0,62-1,32)	0,605
Los profesionales que se ven involucrados en un evento adverso, si lo desean, tienen acceso a la ayuda psicológica del hospital para reducir el impacto en ellos.	De acuerdo	1		1	
	En desacuerdo	1,97 (1,49-2,60)	0,000	1,75 (1,50-2,04)	0,000
	Neutro	1,55 (1,26-1,90)	0,000	1,47 (1,40-1,54)	0,000
He conocido casos de profesionales que han estado en mal estado emocional debido a un evento adverso de un paciente.	No	1		1	
	Sí	2,52 (1,84-3,45)	0,000	1,81 (1,36-2,40)	0,000
He conocido casos de eventos adversos con graves consecuencias para uno o varios pacientes.	No	1		1	
	Sí	2,98 (1,94-4,57)	0,000	1,93 (1,36-2,75)	0,000
Personalmente, he tenido que informar a un paciente que ha sufrido un evento adverso.	No	1		1	
	Sí	2,11 (1,64-2,72)	0,000	1,68 (1,32-2,13)	0,000

RPa: razones de prevalencia ajustadas usando un modelo lineal generalizado con distribución binomial, función de enlace logarítmica y errores robustos ajustados por el área; IC: intervalo de confianza; p: estadístico Z del modelo multivariado; RPc: razones de prevalencia crudas obtenidas a partir de las tablas de contingencia; IC: intervalo de confianza; p: prueba de ji al cuadrado

## Discusión

En las instituciones de salud de todo el mundo se presentan a diario eventos adversos, y “uno de cada diez pacientes que ingresa a un hospital sufre algún tipo de daño a causa de la atención” ^6^, lo que explica que sea común que los profesionales de la salud se vean expuestos a estos en algún momento de su carrera. En este sentido, se encontró que casi la totalidad de los participantes manifestó haber conocido en los últimos cinco años incidentes que podrían haber causado daño a un paciente dentro de la institución, lo que coincide con un estudio realizado en España en el que el 86,3 % de los participantes manifestó haber presenciado en algún momento un incidente ^9^; con esto se constata que los problemas de seguridad en la atención de los pacientes se presentan en diferentes países del mundo.

Dado que los eventos adversos son comunes en las instituciones de salud, la cantidad de profesionales que pueden verse expuestos a estos es grande. En el presente estudio, el 44,4 % de los participantes manifestó haberse visto involucrado en uno de ellos en algún momento. En una revisión de la bibliografía, se encontraron cifras más altas en otros estudios: en uno español fue del 57,8 % ^9^, en otro realizado en hospitales mentales de Bélgica fue del 73,2 % ^15^ y, en Holanda, se reportó en el 2019 que el 85,6 % de profesionales que alguna vez se había visto involucrado en un evento adverso en el curso en su carrera ^16^.

La falta de preparación para afrontar una situación estresante como lo es un evento adverso hace que el personal implicado sienta temor de comunicarlo al paciente o a su familia, tal como se evidenció en un estudio danés en el que así lo manifestaron más de la mitad de los participantes ^17^. El temor a afrontar las consecuencias legales puede ser otra de las barreras para que los profesionales comuniquen los eventos adversos al paciente o a su familia; en un estudio realizado en Estados Unidos, los participantes expresaron que este era el principal obstáculo para notificar un evento adverso ^10^. En el presente estudio, tres de cada cuatro personas dieron una puntuación de 7/10 a su temor de afrontar las consecuencias legales de los eventos adversos.

La comunicación asertiva es pilar fundamental en las relaciones con las demás personas, pero la habilidad para comunicarse con otros, especialmente en situaciones difíciles, puede requerir de una formación específica. Cuando ocurre un evento adverso, el profesional de la salud puede verse emocionalmente afectado, lo que podría dificultar su comunicación con el paciente y su familia. En este sentido, casi la totalidad de los participantes manifestó su deseo de recibir capacitación en torno a cómo informar a un paciente sobre un evento adverso, como también lo expresaron en el estudio español el 89,9 % de los participantes ^9^.

Los eventos adversos pueden producir efectos negativos en los profesionales que se ven involucrados. El 44 % de los participantes manifestó sentimientos como víctimas secundarias, es decir, el 99,5 % de los directamente involucrados manifestó sentirse como una segunda víctima. En este mismo orden de ideas, el 72,5 % de los participantes del área hospitalaria en el estudio español manifestó haberse sentido víctima secundaria de un evento adverso en algún momento ^9^. Asimismo, en un estudio realizado en el Johns Hopkins Hospital en Estados Unidos, así lo manifestaron más de la mitad de los participantes ^18^. En el estudio *Boston Intraoperative Adverse Event Surgeons Attitude* (BISA), realizado en cirujanos de los Estados Unidos, se encontró que el 84 % de los participantes se habían sentido víctimas secundarias de un evento adverso en algún momento ^10^, lo que demuestra cuán común es el fenómeno, incluso en diferentes contextos clínicos y sociales.

Las principales manifestaciones de los profesionales como víctimas secundarias de los eventos adversos en el presente estudio, fueron desconcierto, dificultad para concentrarse en el trabajo, cansancio, ansiedad, rememoración continua del evento y dudas sobre las decisiones que debían tomar. En otros estudios, también se han reportado sentimiento de culpa, ansiedad, rememoración del evento, cansancio, insomnio, dudas sobre las decisiones que se toman, tristeza, vergüenza y miedo a perder la reputación ^9^^,^^10^^,^^19^.

La percepción sobre la probabilidad de un evento adverso de consecuencias graves para el paciente es más alta entre las personas que se han visto directamente involucradas en uno, comparadas con las que no. Esto se evidenció en un estudio español, en el que, además, esta percepción fue menor en las personas que menos reportaron sentirse víctimas secundarias ^9^. Ello demuestra que el haber estado expuesto directamente a un evento adverso y haberse sentido una víctima secundaria de este, aumenta el nivel de conciencia sobre la posibilidad de que ocurran nuevos eventos, lo que podría ser un factor a favor de la toma de medidas preventivas por parte del personal.

El apoyo a las víctimas secundarias de los eventos adversos se convierte en un factor importante en el momento de mitigar sus efectos en los trabajadores de la salud; sin embargo, en el presente estudio, aquellas personas que se habían visto directamente involucradas en uno tenían una percepción desfavorable sobre el apoyo psicológico brindado por la institución. En esto coincide un estudio italiano sobre este mismo problema, en el que todos los participantes habían estado expuestos a un evento adverso y el 85 % manifestó estar insatisfecho con el apoyo recibido de parte de la institución y de su equipo de trabajo ^19^. No obstante, en un estudio danés realizado con obstetras y parteras, el 78,1 % de los participantes manifestaron conocer hasta cierto punto cómo acceder al apoyo emocional brindado por la institución en estos casos ^17^; además, en un estudio en hospitales mentales de Bélgica, el 95,3 % de los participantes manifestaron haber recibido apoyo de su equipo de trabajo ^15^.

La principal limitación del presente estudio fue la inclusión exclusiva de personal de las áreas de medicina y enfermería, ya que ello impide conocer la situación de otras profesiones con respecto al tema. De todas maneras, el estudio es la puerta de entrada a futuras investigaciones en el contexto local, acerca del impacto a corto, mediano y largo plazo de los eventos adversos en los trabajadores de la salud, así como de la manera correcta de informar al paciente y a su familia, la capacidad de los trabajadores de la salud para afrontarlos y la forma más adecuada de brindarles apoyo, con el fin de mitigar su impacto en ellos.

A modo de conclusión, puede decirse que es común que los trabajadores de la salud se vean expuestos a eventos adversos, bien sea directamente o por medio de la experiencia de otros compañeros de trabajo. Por lo general, dichos eventos tienen efectos negativos sobre los profesionales implicados, generándoles tristeza, ansiedad y dudas constantes sobre las decisiones que toman, entre otros. El comunicar un evento adverso a un paciente o a su familia no es una tarea fácil para el personal implicado, y el temor a lo que pueda ocurrir después se convierte en una barrera para la comunicación efectiva en ese momento. Por ello, la capacitación del personal de la salud en torno a la comunicación asertiva en casos estresantes como este, puede cambiar su forma de afrontar la situación.

El apoyo que reciben los profesionales de salud después de verse involucrados en un evento adverso, es importante para mitigar su impacto en ellos, sin embargo, aún falta mucho camino en la preparación de las instituciones en torno a la manera adecuada de brindar dicho apoyo a las víctimas secundarias. El temor del personal asistencial a notificar los eventos adversos podría resultar en su subregistro y, por ende, en menos oportunidades de aprendizaje y de mejora en las instituciones de salud; por lo tanto, brindar las herramientas necesarias al personal de la salud para un adecuado reporte de dichos eventos y una correcta comunicación con la familia y el paciente en esos momentos, podría mejorar la cultura de seguridad del paciente a nivel institucional, ofreciendo un ambiente propicio para que el personal vea tales eventos como una oportunidad de aprendizaje y no como una situación que provoca castigo.

Por otro lado, un profesional con tristeza, ansiedad y dudas sobre las decisiones clínicas que toma, tiene más probabilidades de verse involucrado en un evento adverso, de allí la importancia de mitigar este tipo de sentimientos en las víctimas secundarias, con el fin de brindarles bienestar y, a la vez, mejorar la seguridad del paciente.
